# Preheated restorative composite resin for luting ceramic laminate veneers: An optimized technique report

**DOI:** 10.4317/jced.60068

**Published:** 2023-02-01

**Authors:** Rogério-Luiz Marcondes, Rafael-Ratto Moraes, Jefferson Pereira, Marco-Aurélio de Carvalho

**Affiliations:** 1 PhD, Private Practice, Curitiba-PR, Brazil; 2 PhD, Postgraduate Program in Health Sciences, University of Southern Santa Catarina-SC, Brazil; 3 PhD, Graduate Program in Dentistry, Federal University of Pelotas-RS, Brazil; 4 PhD, Postgraduate Program in Dentistry, Evangelical University of Goias-GO, Brazil

## Abstract

Resin cements are traditionally used to lute ceramic laminate veneers due to their lower viscosity, which facilitates a fast restoration seating. However, resin cements have lower mechanical properties compared to restorative composite resins. Thus, restorative composite resin is an alternative luting agent with lower marginal degradation as a potential advantage for clinical longevity. This article presents an application of preheated restorative composite resin for adhesive luting of laminate veneers with a predictable clinical technique for seating and marginal quality. By addressing important factors that influence film thickness, the predictable presented workflow should overcome this major concern when luting with restorative composite resin, therefore enabling the benefits of using a restorative material with better mechanical properties without the drawback of higher film thickness. Considering the clinical evidence that the adhesive interface between the dental substrate and restoration is the weak link of adhesive indirect restorations, bonding the restoration with preheated restorative composite resins (PRCR) may provide an interface filled with a restorative resin material, presenting optimized mechanical properties.

** Key words:**Resin cements, ceramic laminate veneers.

## Introduction

Bonding of indirect restorations, known as adhesive cementation, comprises one of the most critical steps of adhesive treatment and responds to the majority of clinical failures of indirect restorations reported in literature (1). The main causes of failure reported for indirect restorations are marginal discoloration, marginal degradation, and debonding of the restoration (1). Thus, there is a constant need for better marginal adaptation while attaining film thickness below 120 µm (2), although clinical studies found marginal discrepancies in indirect restorations between 100 and 315 µm (3).

The gap between indirect restorations and tooth surfaces filled by luting material is known as area of adhesive continuity. A thick line of exposed cement could over time be subject to sorption, surface degradation, and wear, leading to marginal ditching and discoloration (4). Even tooth brush abrasion can lead to marginal ditching influenced by dentifrice abrasiveness, tooth brushing force, and direction of the bristles (5). When subject to tooth brushing, resin cements with larger filler particles have shown increased wear than those with smaller particles (5). In addition, resin cements showed greater marginal degradation than resin composites (6).

Composite resin cements (CRC) are traditionally used for luting indirect restorations due to the simplicity of application associated with their lower viscosity, which enables fast restoration seating. However, PRCR have been increasingly used for bonding non-retentive partial restorations (7). The benefits that would justify the use of composites are related to lower marginal degradation, greater color stability, and improved mechanical strength (6,8). Preheating of restorative composites with appropriate rheological properties enables its predicTable use for bonding indirect restorations without jeopardizing film thickness (9,10). Associated with preheating, the use of ultrasonic devices favors a faster excess removal during the seating of restorations and may aid in reducing the luting agent film.

Recent systematic review (11) have shown an increase in film thickness when using PRCR. Even though the clinical marginal discrepancies found in indirect restorations range between 100 and 315 µm (3), film thickness is still a concern when using PRCR for luting. Many important factors may influence on the film thickness, such as the suitable rheological property of composite resin, seating during optimal temperature and viscosity, the use of ultrasound device and light-curing under pressure (12). The studies included on the systematic literature reviews (13,14) failed to address those critical factors when luting with PRCR. Therefore, it is necessary to present the optimized technique for luting with PRCR, granting adequate film thickness as found is some studies that observed those critical factors (9,10).

Considering the clinical evidence that the adhesive interface between the dental substrate and restoration is the weak link of adhesive indirect restorations, bonding the restoration with PRCR may provide an interface filled with a restorative resin material, presenting optimized mechanical properties. Therefore, an area of adhesive continuity that is more resistant to degradation and staining is expected to provide improved restoration prognosis (8). This article reports an optimized and comprehensive clinical technique for luting ceramic laminate veneers with preheated restorative composite resin, ensuring better film thickness.

## Case Report

**Figure 1:**
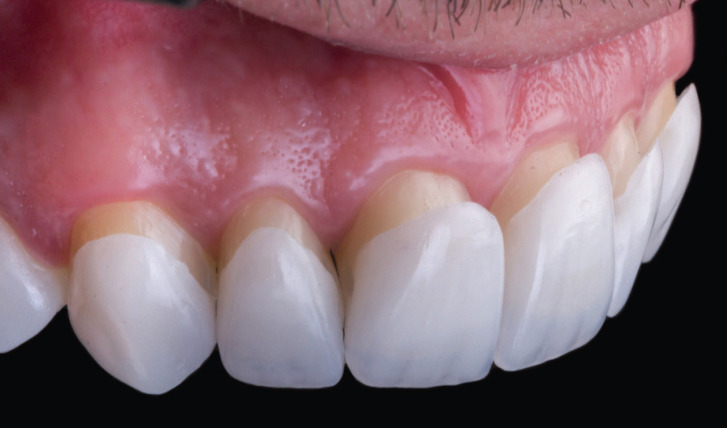
After removal of provisionals, dry try-in is performed to assess the fitting of laminate veneers, including marginal adaptation and insertion axis of each restoration.

**Figure 2:**
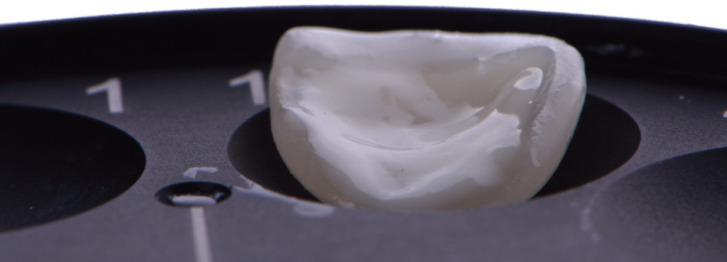
Ceramic laminate veneer was loaded with restorative composite resin that was preheated on the first warmer and then placed and covered in the second warmer for reheating back to 155°F/68°C.

**Figure 3:**
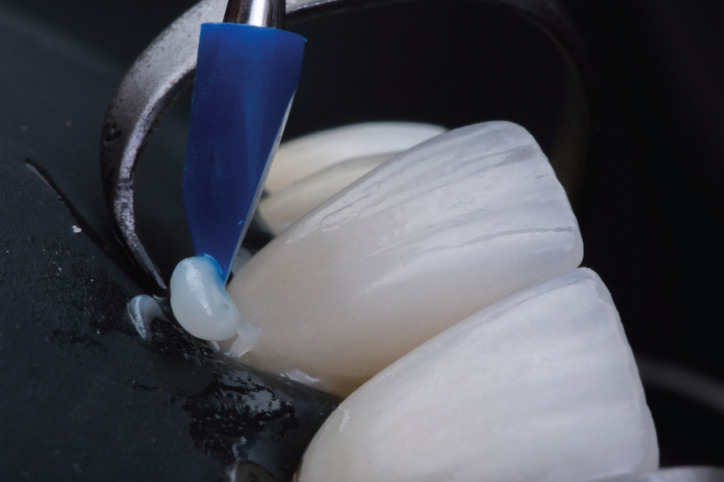
Removal of restorative composite excesses right after initial seating of the veneer. Excess removal is easy to perform because the preheated composite acquires higher viscosity within seconds after seating.

Figures 1 to 3 illustrate the technique, which was carried out using the following clinical steps:

1. Ceramic laminate veneers were prepared for ten maxillary teeth to recover incisal length and esthetic enhancement of the smile.

2. Composite warmers are set at 155°F/68°C and composite syringes/compules are placed for a 10-minutes warm-up 

3. Removal of provisional restorations, dry and wet try-in of the laminate veneers on the prepared teeth (Fig. 1).

4. The operative field was isolated with rubber dam for moisture control and proper access to margins.

5. Dry try-in of the laminate veneers after rubber dam isolation to ensure correct restoration seating even with a clamp;

6. The intaglio feldspar ceramic surfaces (Creation CC; Willi Geller International GmbH, Meiningen, Austria) were etched with 9.5% hydrofluoric acid for 60 seconds (Porcelain Etchant; Bisco, Schaumburg, USA), cleaned with 35% phosphoric acid for 15 seconds (Ultra Etch; Ultradent, South Jordan, USA), silanated (Bis-Silane; Bisco), and filled with hydrophobic adhesive (OptiBond FL; Kerr, Brea, USA).

7. Compules of restorative composite resin (Estelite Omega, shade BL2, Tokuyama, Tokyo, Japan) were preheated to 155°F/68°C for 10 minutes in the first warmer (CalsetTM, AdDent Inc. Danbury, CT, USA). The composite was applied to the veneers with Centrix syringe. The veneers loaded with preheated composite resin were positioned on the tray of the second warmer also at 155°F/68°C and covered for reheating during tooth preparation conditioning (Fig. 2).

8. Tooth preparation conditioning: enamel was air-abraded with 50 µm aluminium oxide particle (Cobra Aluoxyd, Renfert, Hilzingen, Germany) and then etched with 35% phosphoric acid gel for 30 seconds, washed, dryed, and the same adhesive was applied on the preparation.

9. Loaded restorations were removed from second warmer and immediately and cautiously positioned on prepared teeth. Seating by hand pressure was applied.

10. Initial removal of composite resin excesses (Fig. 3).

11. Ultrasonic activation applied over ceramic with ultrasonic unit (Dentsurg; CVDentus, São José dos Campos, SP, Brazil) and polyacetal tip at 40% power and dry to further increase composite flowability and reduce film thickness. More excesses are removed on this stage.

12. Light-curing under pressure for 60 seconds on each face (20 seconds × 3, with intervals of 10 seconds between applications for cooling down the tooth);

13. Additional 10 seconds marginal light-curing using water-soluble gel to reduce oxygen-inhibited layer;

14. Finishing with scalpel blades and polishing with diamond polishers (D.Fine; Clinician’s Choice, New Milford, CT).

## Discussion

Preheated restorative composite resin may be considered an excellent clinical option for luting ceramic laminate veneers due to its improved mechanical properties (6,8), but care should be taken when choosing this technique as it needs training and adequate apparatus, and sequence to achieve adequate temperature for decreasing viscosity and allowing predicTable seating (12). Proper seating of restorations may not be achieved if important factors are not considered when luting with PRCR such as proper composite resin selection; seating during optimal luting agent temperature and viscosity; the use of ultrasound device (optional); and light-curing under pressure. Neglecting such important factors is the reason for thicker films found in many *in vitro* tests (11-14). Therefore it is crucial to properly address the best clinical workflow when using PRCR, even though this technique has been used for decades.

Not all restorative composite resin is suitable for luting. Some composite resins, even after heating, are not adequate for the viscosity decrease needed for luting (9). Choosing a composite with inadequate rheological quality may prevent optimal flowability and proper seating of restorations. Some restorative composites have been shown to be contraindicated as luting agent, as they provide unaccepTable film thickness (9,14). In contrast, there are composites resins with a high amount of inorganic fillers, excellent mechanical and optical properties that could be indicated for luting. A proper selection would not compromise the restoration seating (10), provide adequate film thicknesses (9), reaching lower marginal degradation, greater color stability, and greater mechanical strength (6,8).

The heating technique also plays an important role in providing adequate viscosity. Specific composite warmers should be used, and hot water bath should be avoided as it is more time consuming and time is associated with temperature decrease and viscosity increase (9). Optimally, two composite warmers should be used at 155°F/68°C. The first warmer is used for preheating the composite compules/syringes. During the accommodation of the composite in the intaglio surface of the restoration, heat will be lost and viscosity will increase considerably after 15 seconds (9). For that reason, the use of a second warmer is advised, for reheating the restoration loaded with composite up to 155°F/68°C meanwhile the tooth preparation is conditioned. Thus, between the removal of the loaded restoration at 155°F/68°C from the second warmer and seating on tooth preparation, less than 15 seconds will pass, ensuring the lowest viscosity and optimal seating.

Associated with the preheating of the composite, the use of ultrasonic device over the ceramic allows more accurate fitting of these restorations, reducing film thickness, thus this instrument should be considered when using the technique. To ensure the final restoration seating, initial light-curing should be always carried out under proper pressure. Finger or padded composite spatulas can be used to ensure proper seating during light-curing. This ensures the restoration will not dislocate, which would generate marginal misfit and greater need for occlusal adjustments (14). When proper pressure is applied during light curing, adequate seating is achieved, regardless of using resin cement or preheated restorative composite resin (10).

Some situations should be avoided when luting with PRCR: composite resins that do not significantly decrease its viscosity (inadequate rheological property); inadequate composite temperature during seating (less than 155°F/68°C); and light-curing without proper pressure will increase film thickness, jeopardizing restoration seating as reported in recent literature reviews (11,12). In addition, by not reaching the ideal flowability, the risk of thin restorations fracture during seating will increase. If proper material and technique are used, restorative composite resin could be used as luting agent with adequate seating (10) and clinical success (8).
